# Potential antibacterial effects and transcriptomic analysis of a novel reversible photoacid-based crystalline coordination polymer

**DOI:** 10.3389/fmicb.2025.1624377

**Published:** 2025-07-14

**Authors:** Chenhua Zheng, Yaying Zheng, Binjie Wu, Yuyan Zheng, Shuye Yu, Rui Qiu, Wanling Chen, Xin Chen, Longze Li, Jianzhen Liao, Fen Hu

**Affiliations:** ^1^Key Laboratory of Gastrointestinal Cancer, Ministry of Education, School of Basic Medical Sciences, Fujian Medical University, Fuzhou, China; ^2^Department of Pharmacy, Fujian Children’s Hospital (Fujian Branch of Shanghai Children’s Medical Center), College of Clinical Medicine for Obstetrics and Gynecology and Pediatrics, Fujian Medical University, Fuzhou, China; ^3^College of Materials and Chemical Engineering, Pingxiang University, Pingxiang, China

**Keywords:** photoacid, clinical pathogens, inhibition rate, SEM, transcriptomic analysis

## Abstract

**Introduction:**

With the increasing prevalence of antibiotic resistance, the development of novel antibacterial materials is crucial to combat clinically relevant pathogens. This study comprehensively investigated the antibacterial properties and underlying mechanisms of a novel reversible photoacid-based crystalline material.

**Methods:**

The antibacterial efficacy of the material was evaluated against six clinically relevant pathogenic bacteria, including multidrug-resistant strains. The inhibition rates were determined, and scanning electron microscopy (SEM) was used to observe the effects on cell surface integrity. Transcriptomic analysis was conducted to elucidate the underlying antibacterial mechanisms.

**Results:**

The material exhibited broad-spectrum antibacterial activity, with higher sensitivity toward Gram-negative bacteria. Blue light irradiation significantly enhanced its antibacterial efficacy. SEM revealed that the material disrupted cell membrane integrity, leading to cell death. Transcriptomic analysis showed that the material inhibited bacterial protein synthesis, disrupted cell membrane protein synthesis, and downregulated oxidative stress-related genes, causing ROS accumulation and inhibiting cell growth.

**Discussion:**

These findings provide a theoretical basis for the potential clinical application of this material as a new antibacterial agent. The material’s ability to enhance antibacterial efficacy through light irradiation and its broad-spectrum activity suggest it could be a valuable tool in combating antibiotic-resistant pathogens. Future research should focus on further exploring the antibacterial mechanisms and evaluating the material’s safety and efficacy in clinical settings.

## 1 Introduction

The escalating misuse of antibiotics has given rise to antibiotic resistance, progressively diminishing the efficacy of conventional antibacterial and bactericidal agents. As globalization accelerates, antibiotic resistance has emerged as a significant global health threat, underscoring the urgent need for the continuous development of novel antibacterial and bactericidal agents (Pulingam et al., 2022; Mendelsohn et al., 2023). Antimicrobial resistance is responsible for approximately 700,000 deaths annually, with the economic burden in the United States alone exceeding $20 billion per year. Projections indicate that by 2050, in the absence of effective interventions, the annual death toll could soar to 10 million (Kraker et al., 2016). The ineffectiveness of most antibiotics against “superbugs” highlights the critical need for innovative strategies to inhibit bacterial virulence and biofilm formation, thereby preventing pathogen invasion. The development of bactericides with mechanisms distinct from traditional antibiotics represents one of the most promising alternative approaches to overcoming antibiotic resistance. While some non-antibiotic antibacterial materials have been developed (Shaomin et al., 2022; Shu et al., 2021; Baig et al., 2023; Banti and Hadjikakou, 2022), their number remains insufficient. Consequently, the research and development of novel antibacterial materials are of paramount importance. These materials have the potential to serve as new weapons in combating microbial infections, thereby reducing infection risks, enhancing medical care quality, safeguarding public health, boosting economic development, and addressing the global challenges posed by microbial infections. Against this backdrop, researchers are actively exploring various advanced materials with innovative antibacterial properties, including nanoparticles, hydrogels, and surface coatings, among which photoacid materials have garnered considerable attention (Bassegoda et al., 2018).

Photoacids are molecules that upon exposure to light, can undergo acid–base transitions and inducing changes in the pH environment (Bae et al., 2022), thereby altering the electronic and optical properties of the materials. Light irradiation can trigger proton dissociation, enabling spatiotemporal control of proton transfer within the photoacid system. This non-contact triggering method and the reversible proton transfer process offer significant advantages for the application of photoacid-based materials across various fields. In recent years, the application of photoacid materials in the biomedical field has garnered considerable attention due to their exceptional clinical potential (Sun et al., 2024; Jiang et al., 2025). An increasing number of novel photoacid materials have been reported, with diverse applications in sensors (Tse et al., 2022), molecular switches (Zhang et al., 2018), antibacterial applications (Hu et al., 2017), supramolecular assembly (Wang et al., 2022), and photo-chromism (Zhang et al., 2018), among others. The most prevalent photoacids include spiropyrans, tricyanofurans, and pyran derivatives. Photoacid systems are categorized into liquid and solid states. Solid materials, in particular, benefit from the functionality of covalent photoacids, which allow modification of their electronic and optical properties upon light exposure without the need for additional photoacid solutions. Additionally, these materials can induce local changes in proton concentration both within and surrounding the solid material (Liao, 2017). Modifying this specific microenvironment is a prerequisite for acquiring certain characteristics and applications of photoactive materials. Consequently, functional polymers and solid materials based on metastable photoacids have become focal points of research for chemists in recent years (Ke et al., 2020).

In a previous study, a photoacid-based crystalline material [CaNa₂(HPTS)(NO₃)(H₂O)₅] (referred to as Compound 1) was successfully synthesized via a solvothermal method (Liao et al., 2023). This material, composed primarily of 8-Hydroxypyrene-1,3,6-trisulfonic acid trisodium salt (HPTS) (Sigma-Aldrich, United States), exhibited a dense and regular structure. Compound 1 was constructed through the self-assembly of a highly metastable HPTS photoacid, driven by coordination with an appropriate metal cation Ca^2+^ (Figure S1). This material not only enhanced light absorption efficiency but also displayed significant red-shifted spectral light absorption, covering the entire visible region and extending into the near-infrared domain, with excitation achievable through blue light (Figure S2). The ordered arrangement of HPTS molecules, combined with strong hydrogen bond interactions between proton donors and acceptors, established an effective pathway for excited-state proton transfer and charge transfer within the crystal matrix. This maintained and even enhanced the photoacid properties of the material (Figure S3).

To investigate the antibacterial properties of Compound 1, we selected six clinically relevant pathogenic bacteria as test strains. We examined the effect of this material on the inhibition ratio (IR) of these six clinical pathogens. The impact of this material on the cell membranes of different test bacteria before and after treatment was observed using a scanning electron microscope. To investigate the antibacterial mechanism, MRSA was chosen as a model for transcriptomic profiling due to its clinical relevance and distinct response to Compound 1. This study aims to evaluate the antibacterial potential of Compound 1 against six clinically relevant pathogenic bacteria and elucidate its antibacterial mechanism through transcriptomic analysis, thereby providing new possibilities and a theoretical basis for the development of novel antibacterial agents.

## 2 Methods

### 2.1 Inhibition ratio detection

We selected six clinically relevant pathogenic bacteria as test strains (the six strains were kindly provided by Professor Hang Yang from the Wuhan Institute of Virology, Chinese Academy of Sciences), including Gram-negative bacteria such as *Escherichia coli* BL21 (DE3) and *Pseudomonas aeruginosa* ATCC9027, and Gram-positive bacteria such as *Staphylococcus aureus* ATCC6538 and *Bacillus subtilis* CMCC63501. Additionally, we included a clinically multidrug-resistant *Pseudomonas aeruginosa* (MDR-PA) P53 and a clinically methicillin-resistant *Staphylococcus aureus* (MRSA) ATCC43300. To induce darkening and maintain the initial state of proton release, Compound 1 was first exposed to blue light. It was then dissolved in sterile LB (Luria-Bertani Broth) liquid medium (Huankai, Guangdong, China) and added to a bacterial suspension that had been grown to the logarithmic phase. Each tube contained 3 mL of bacterial suspension, with the final concentrations of Compound 1 set at 0 mg/mL, 0.005 mg/mL, 0.01 mg/mL, 0.025 mg/mL, 0.05 mg/mL, 0.1 mg/mL, 0.25 mg/mL, 0.5 mg/mL, 1.0 mg/mL, and 2.0 mg/mL. The bacterial suspensions were incubated 16 h in a 37°C incubator (DNP-9272, Shanghai Jinghong, China) and irradiated with 3 W LED blue light (wavelength: 460–465 nm, 5 cm distance, 16 h continuous exposure). Subsequently, each group’s bacterial suspensions underwent a 10-fold serial dilution using phosphate-buffered saline (PBS, 10 mM, pH 7.4). After appropriate dilution, the suspensions were spread onto LB agar plates for cultivation and counting. The inhibition ratio was determined by dividing the number of colony-forming units (CFU). IR was calculated using the following equation:


$$\mathrm{IR}=\frac{C_{i}-C_{a}}{C_{i}}\times 100$$

Where C_a_ is the CFU of the experimental group treated with blue light (or not) in the presence of Compound 1, and C_i_ is the CFU of the control group without compounds.

### 2.2 Effect of blue light and ligand on IR

*Escherichia coli* was used as the target bacteria. Since HPTS serves as the primary component for synthesizing Compound 1, it was included as a control group to exclude its potential influence on the antibacterial performance of Compound 1. To comprehensively evaluate light-dependent effects, the cultivation process was divided into three distinct treatment groups: (1) Compound 1 with blue light irradiation, (2) Compound 1 without blue light irradiation, and (3) HPTS with blue light irradiation. The concentrations of Compound 1 and HPTS added were 0 mg/mL, 0.1 mg/mL, 0.25 mg/mL, 0.5 mg/mL, 1.0 mg/mL, and 2.0 mg/mL, respectively. The culture conditions and the method for detecting the IR were consistent with those described previously.

### 2.3 Scanning electron microscopy

Six clinically relevant pathogenic bacteria were treated with Compound 1 at a concentration of 0.1 mg/mL and cultured 16 h under blue light exposure. Bacterial samples that were not treated with Compound 1 served as controls. The samples were centrifuged (5810R, Eppendorf, Germany) at 8,000 rpm for 10 min, and the supernatant was discarded. The pellets were then washed with PBS. Subsequently, the washed samples were placed in 2.5% glutaraldehyde for fixation for 3 h. After fixation, the samples were washed three times with PBS, centrifuged, and the supernatant was discarded again. The processed samples were then subjected to gradient dehydration in ethanol solutions of 50, 70, 80, 90, and 100%, with each dehydration step lasting 15 min. The 100% ethanol dehydration was repeated twice, each for 15 min. The samples were then placed in a mixture of ethanol and tert-butanol (1:1) for 15 min, followed by discarding the supernatant. Pure tert-butanol was used to replace ethanol twice, each for 15 min. The bacteria-tert-butanol suspension was mixed well and dropped onto a cover slip. The cover slip with the sample was frozen in a − 80°C freezer and then placed in a freeze dryer for lyophilization (BTP-3XL00X, SP Scientific, United States). After the samples were thoroughly dried, the cover slip was adhered to a sample holder coated with conductive tape. The samples were then sputter-coated with gold and observed under a scanning electron microscope (SU-80100, Hitachi, Japan). All SEM images were systematically acquired from a minimum of five randomly selected fields per sample. For comprehensive analysis, we examined at least 50 bacterial cells per treatment group across a magnification range of 6,000 × to 25,000 × .

### 2.4 Transcriptome sequencing

Total RNA was extracted from MRSA in both the control group and 0.1 mg/mL Compound 1-treated group, the experiment utilized the TruSeq TM Stranded Total RNA Library Prep Kit (Illumina, Cali-fornia, United States) to construct the library. Sequencing was conducted using the NovaSeq X Plus platform (Illumina, California, United States). Raw reads were filtered by cutadapter (version 1.11) to remove adapter sequences and low quality reads. The remaining reads were quality checked by fastqc (version: 0.11.5).^1^ Clean reads were mapped to the *S. aureus* ATCC 43300 genome.^2^ Differential expression analysis was performed using the edgeR program (Smyth, 2010). Genes with a p-adjusted value<0.05 and |log2(fold change)| > 1 were considered to be differentially expressed. The sequencing was performed by the Igenebook Bioinformatics Institute (Wuhan, China).

### 2.5 Statistical analysis

All experimental data were derived from three independent biological replicates and were presented as mean ± standard deviation (SD). Statistical analyses were performed using GraphPad Prism software (version 9.0). Differences between treatment groups were assessed by one-way analysis of variance (ANOVA) followed by Dunnett’s *post hoc* test for multiple comparisons to the control group. The threshold for statistical significance were indicated as *p* > 0.05 (ns), *p* < 0.05 (*), *p* < 0.01 (**) or *p* < 0.001 (***).

## 3 Results

### 3.1 Effect of light and ligand on IR

**Figure 1:**
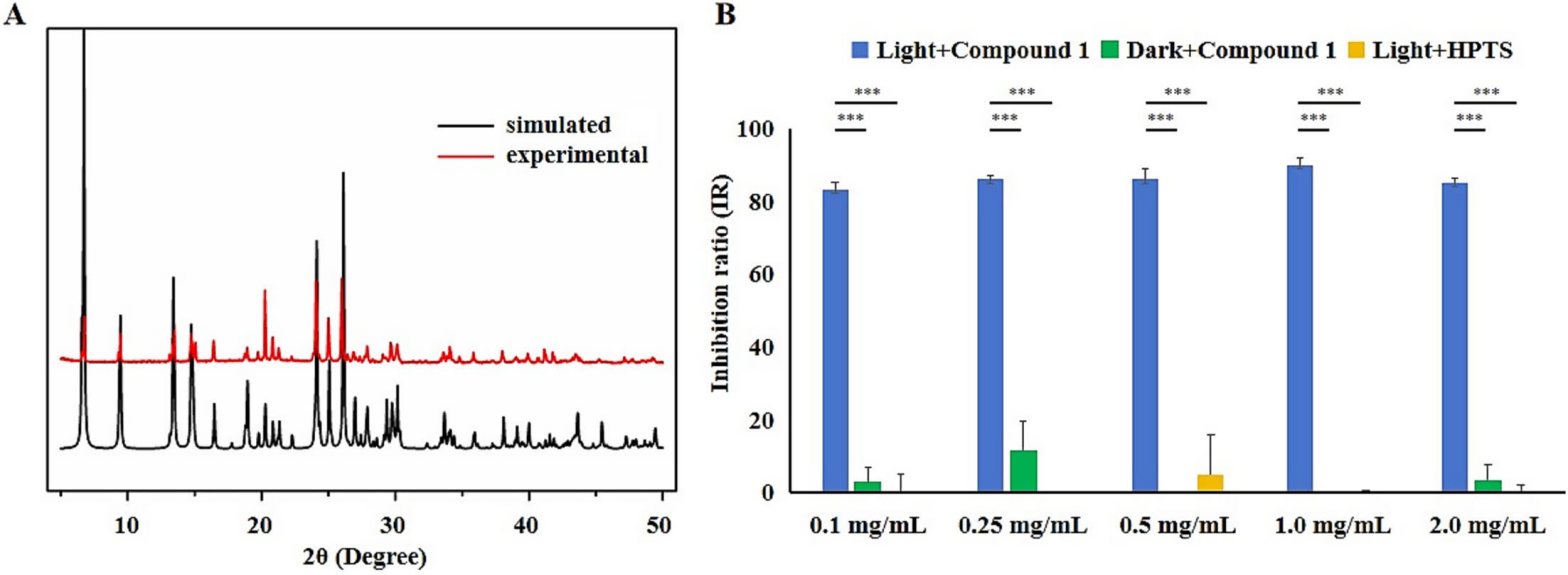
The effects of different treatments on the IR of *E. coli*. **(A)** Comparison of photoacid-based crystal materials and simulated PXRD patterns. **(B)** Blue: blue light irradiation with Compound 1 added; Green: dark conditions with Compound 1 added; Yellow: blue light irradiation with HPTS added. One-way ANOVA–Dunnett test; *p* < 0.05 (*), *p* < 0.01 (**) or *p* < 0.001 (***).

The experimental powder X-ray diffraction (PXRD) pattern of the material matches the simulated spectrum based on single-crystal X-ray diffraction data (CCDC 2269377), confirming that the synthesized Compound 1 has a high purity in the experiment (Figure 1A; Liao et al., 2023). To investigate the effects of blue light irradiation and HPTS on the antibacterial efficacy of Compound 1, three distinct treatment groups were established: Compound 1 with blue light irradiation, Compound 1 in darkness, and HPTS with blue light irradiation. The group treated with Compound 1 under blue light irradiation exhibited a significantly higher IR against *E. coli* compared to the groups treated with Compound 1 in darkness and the groups treated with HPTS under blue light irradiation (Figure 1B). This indicates that blue light irradiation enhances the antibacterial efficacy of Compound 1, while HPTS does not significantly affect its antibacterial performance.

### 3.2 Effect of Compound 1 on the IR of various pathogenic Bacteria

**Figure 2:**
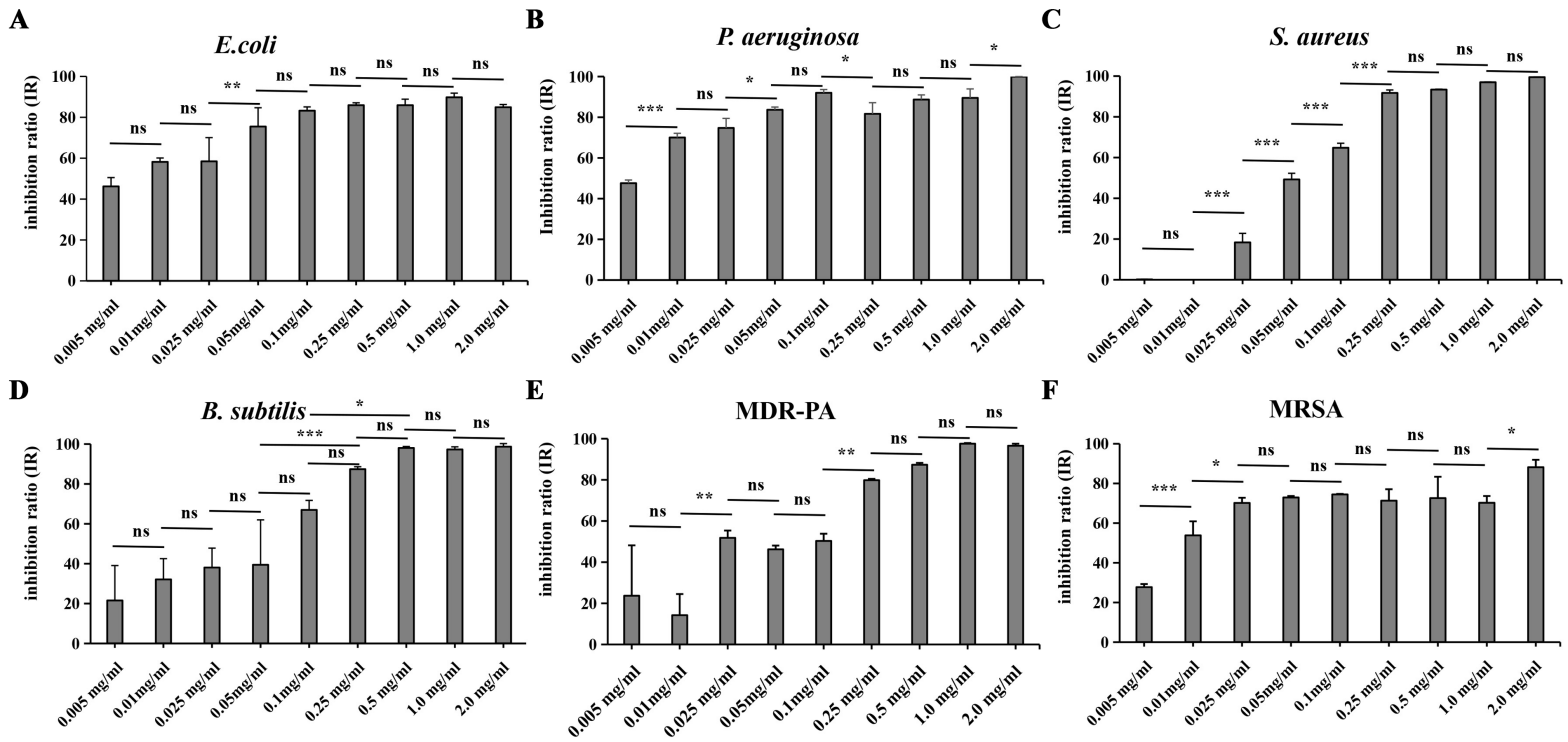
The effect of adding different concentrations of Compound 1 on the IR of six common pathogenic bacteria. **(A)** *E.coli.* **(B)** *P. aeruginosa* **(C)**. *S. aureus* **(D)** *B. subtilis*. **(E)** MDR-PA **(F)**. MRSA. Six strains were added with the Compound 1 at concentrations of 0 mg/mL, 0.005 mg/mL, 0.01 mg/mL, 0.025 mg/mL, 0.05 mg/mL, 0.1 mg/mL, 0.25 mg/mL, 0.5 mg/mL, 1.0 mg/mL, and 2.0 mg/mL when they had grown to the logarithmic phase. Then they were incubated 16 h at 37°C under blue light exposure. The IR was determined for each strain at the various concentrations of Compound 1. *p* > 0.05 (ns), *p* < 0.05 (*), *p* < 0.01 (**) or *p* < 0.001 (***).

To evaluate the antibacterial efficacy of Compound 1 against clinically relevant pathogens, six common pathogenic bacteria were selected for study. As shown in Figure 2, Compound 1 exhibits broad-spectrum antibacterial activity against these strains. With increasing concentration of Compound 1, the inhibition rate against the six strains shows an upward trend. However, when the concentration reaches a certain level, the inhibition rate plateaus. The concentration at which this plateau is reached varies among different strains. When the concentration of Compound 1 was set at 0.01 mg/mL, the IR against *E. coli*, *P. aeruginosa*, and MRSA exceeded 50%. For these three strains the inhibition rates reached a plateau at concentrations of 0.05 mg/mL, 0.1 mg/mL, and 0.025 mg/mL, respectively, these plateau concentrations were lower than those for the other three strains. These results indicate that Compound 1 exhibits higher sensitivity toward *E. coli*, *P. aeruginosa*, and MRSA to the other three strains tested. Upon increasing the concentration of Compound 1 to 1 mg/mL, the IR against *P. aeruginosa*, MDR-PA, *S. aureus* and *B. subtilis* all surpassed 95%. In contrast, the IR against *E. coli* and MRSA were both below 90%.

When compare the IR of two pairs of clinical drug-resistant bacteria and wild-type bacteria we found that, when compared to *P. aeruginosa* and MDR-PA, Compound 1 has a higher IR against *P. aeruginosa* at low concentrations. In contrast, when comparing MRSA to *S. aureus*, Compound 1 have a higher IR against MRSA at low concentrations. However, when the concentration is increased to 2 mg/mL, *S. aureus* achieve complete inhibition, while MRSA only reached an IR of 88%.

### 3.3 The effect of Compound 1 on the cell surface of different strains

**Figure 3:**
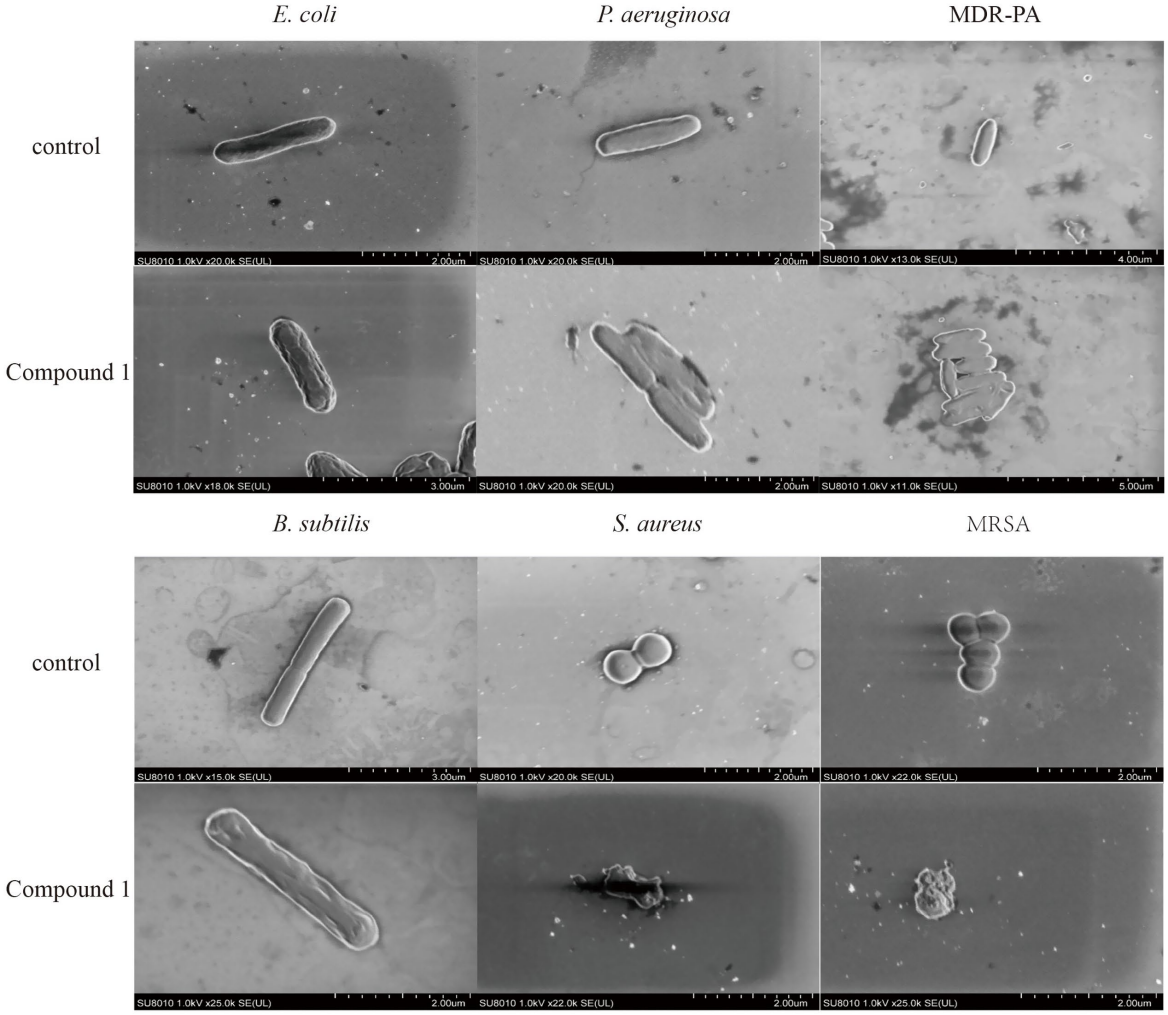
The effect of Compound 1 on cell membrane of different strains. SEM images of *E. coli*, *P. aeruginosa*, MDR-PA, *B. subtilis*, *S. aureus* and MRSA cells after they were the treated with the Compound 1 at a concentration of 0.1 mg/mL, and the control experiment.

As shown in Figure 3, *E. coli* exhibits a short rod shape with a smooth cell surface. After the addition of Compound 1, the cell surface becomes indistinct, with the appearance of shrinkage and inward curving indentations. *P. aeruginosa* also has a short rod shape with a smooth cell wall. Upon the addition of Compound 1, its appearance becomes deformed, with shrinkage and indentations on the surface. The cell surface changes in the MDR-PA strain are similar to those in *P. aeruginosa. B. subtilis* has a rod-like appearance with a smooth cell wall and a visible septum in the middle of the cytoplasm. After the addition of Compound 1, the cell surface becomes deformed, with shrinkage and indentations on the surface. *S. aureus* is spherical with an intact and smooth cell surface. After the addition of Compound 1, the cells increased surface permeability, with pores appearing in the cell wall and cell membrane, leading to the leakage of cellular contents. The morphological changes in MRSA are similar to those in *S. aureus.*

### 3.4 The effect of Compound 1 on the transcriptome of MRSA

**Figure 4:**
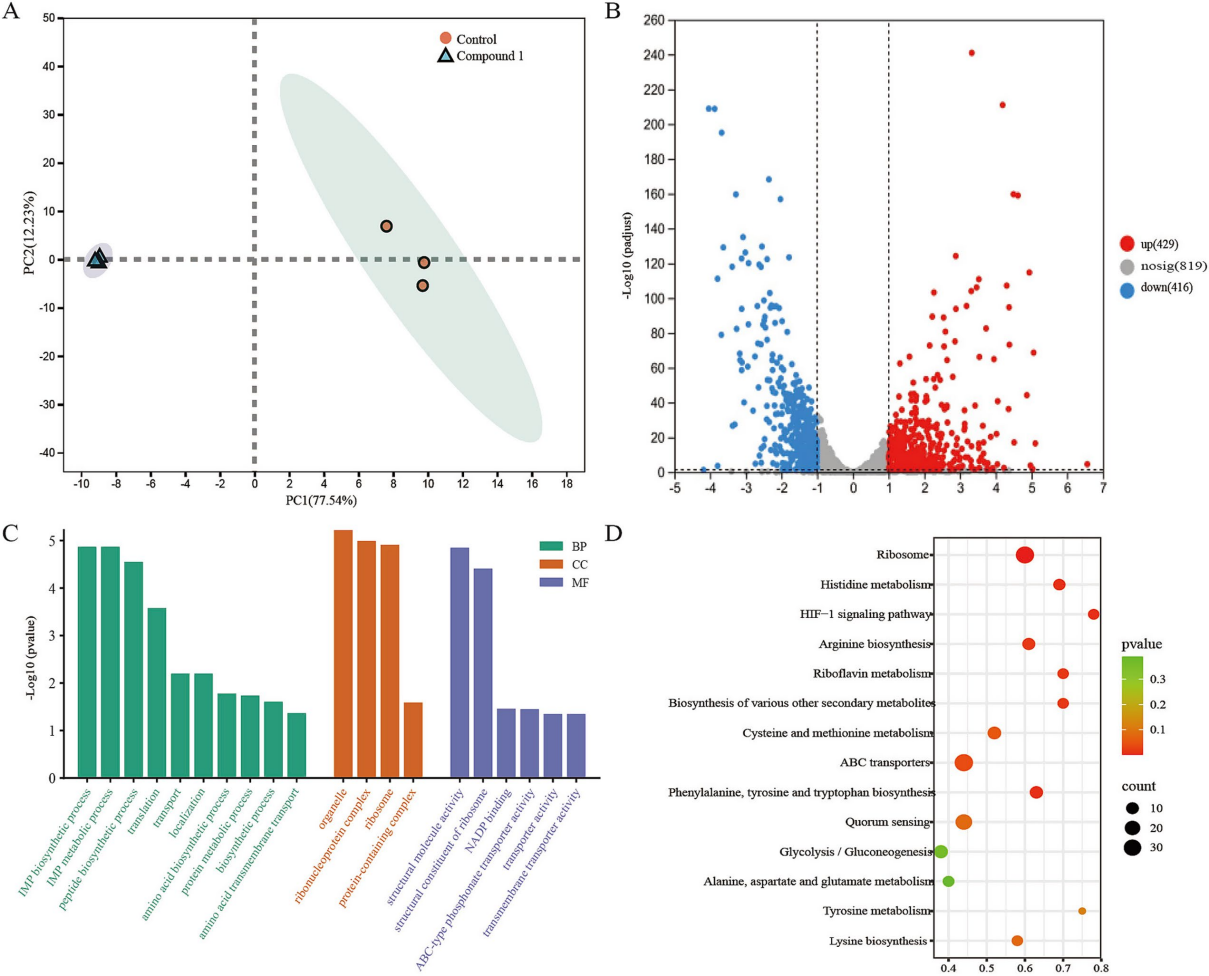
Molecular diversity of MRSA following Compound 1 treatment. **(A)** PCA analysis of RNA sequencing results showing differences in gene expression between control and Compound 1-treated MRSA. **(B)** Volcano plot showing differentially expressed genes between control and Compound 1-treated MRSA. Significance was defined as padjust<0.05 and |log2(fold change)| > 1. **(C)** Representative GO terms after Compound 1 treatment. **(D)** Representative KEGG entries after Compound 1 treatment.

We performed RNA sequencing on the MRSA transcriptome after treatment with 0.1 mg/mL of Compound 1. This analysis aimed to identify differentially expressed genes (DEGs) between the control group and the Compound 1-treated group. Principal component analysis (PCA) of the sequencing data revealed a clear separation in the gene expression profiles between the control and material-treated groups (Figure 4A). A total of 2,365 genes with non-zero variance were used as input variables for the PCA. The first principal component (PC1) accounted for 77.54% of the total variance, while PC2 explained 12.23%. In the material-treated group, a total of 845 differentially expressed genes were identified, including 429 upregulated genes and 416 downregulated genes (Figure 4B). These findings indicate significant changes in gene expression in MRSA following treatment with the Compound 1.

To further explore the functions of these differentially expressed genes (DEGs), we conducted Gene Ontology (GO) analysis to identify the associated biological processes (BPs), cellular components (CCs), and molecular functions (MFs). The results indicated that the BPs were primarily enriched in metabolic processes, biosynthetic processes, translation, and transport. The CCs were mainly enriched in ribonucleoprotein complexes, ribosomes, and protein-containing complexes. The MFs were predominantly enriched in structural molecule activity, structural constituents of the ribosome, NADP binding, and transporter activity (Figure 4C).

To determine which metabolic pathways these DEGs play key roles in, we performed Kyoto Encyclopedia of Genes and Genomes (KEGG) pathway enrichment analysis to further elucidate their biological functions. Following material treatment, pathways such as Ribosome, Histidine metabolism, transporters, and the HIF-1 signaling pathway were significantly enriched, which consistent with our GO enrichment analysis results. This suggests that the addition of the Compound 1 mainly affects MRSA’s material metabolism, energy metabolism, and protein synthesis (Figure 4D).

**Figure 5:**
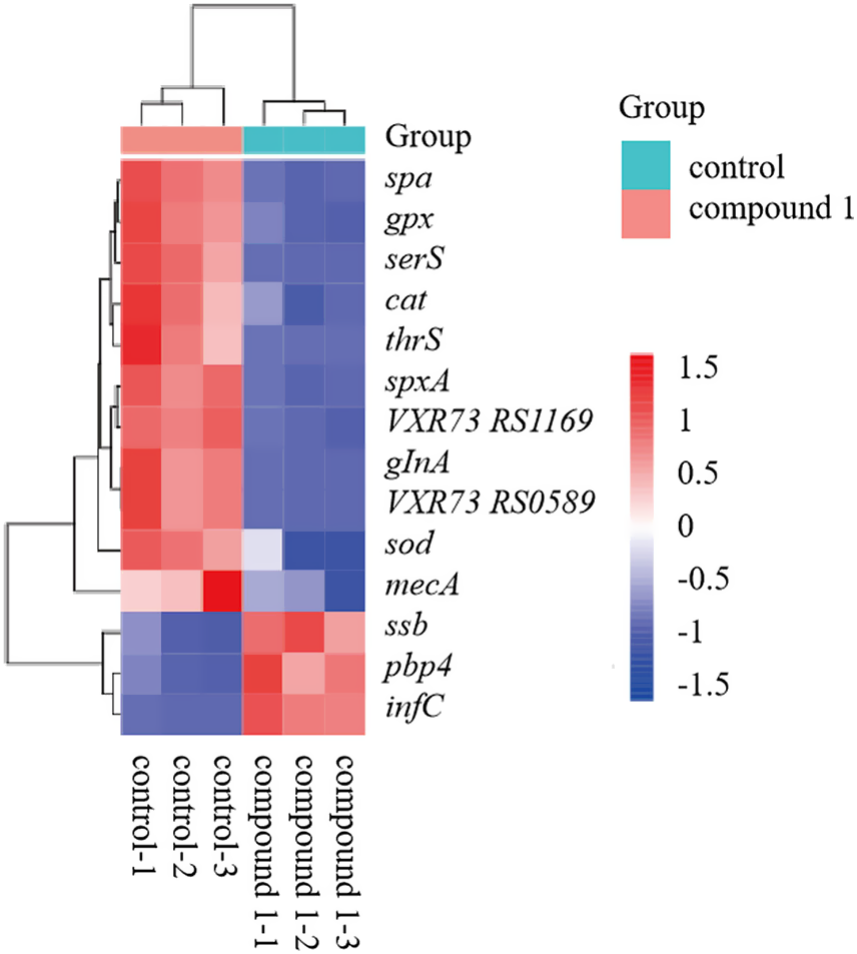
Heatmap showing expression levels of key genes in the control and compound 1-treated groups.

Upon analysis of the DEGs, we observed significant changes in the expression of key virulence factors, genes involved in protein synthesis, and genes related to reactive oxygen species (ROS) response between the control and material-treated groups of MRSA. The heatmap revealed that in the material-treated group, the expression of virulence factor-related genes (*mecA*, *spa*), protein synthesis process-related genes (*glnA*, *VXR73_RS05895 (MerR)*, *spxA*, *serS*, *VXR73_RS11695*, *thrS*), and ROS response-related genes (*gpx, cat, sod*) were downregulated. In contrast, the expression of the translation initiation factor IF-3 (*infC*), single-stranded DNA-binding protein (*ssb*), and penicillin-binding protein PBP4 (*pbp4*) was upregulated (Figure 5). Among the transport proteins, ATP-binding cassette (ABC) transporters and Major Facilitator Superfamily (MFS) transporters were primarily analyzed. In the results, 38 ABC transporters genes were significantly upregulated and 25 ABC transporters genes were significantly downregulated. For the MFS transporters, 8 genes were significantly upregulated and 2 genes were significantly downregulated.

## 4 Discussion

This study demonstrates that Compound 1 exhibits concentration-dependent antibacterial activity against both Gram-positive and Gram-negative pathogens, with particularly strong efficacy against multidrug-resistant strains under blue light irradiation (460–465 nm). Transcriptomic analysis revealed that Compound 1 disrupts bacterial protein synthesis and oxidative stress responses, while SEM observations confirmed significant cell surface damage at effective concentrations (0.1 mg/mL).

These findings align with but extend previous work on photoacid antibacterial mechanisms. Liu et al. (2021) reported that photoresponsive polysulfonates can trigger irreversible broad-spectrum sterilization via ultraviolet light, including Gram-positive *S. aureus* and Gram-negative *E. coli* and *P. aeruginosa*. At a treatment concentration of 2 mg/mL, these materials achieved 100% inhibition. The irreversible photacid reaction, which causes a sustained pH drop, may lead to irreversible damage to cells and their vital activities. Luo et al. (2013) utilized a photacid that can reversibly change the pH by more than two units. Under 470 nm visible light irradiation, colistin combined with the photacid material can kill 99% of MDR-PA. More importantly, this material reduced the minimum inhibitory concentration of colistin against MDR-PA from 8 mg/mL to 0.25 mg/mL. Compared with irreversible photacids, reversible photacids do not produce by-products after light irradiation and do not accumulate H^+^ within cells, which may make them safer for biomedical applications (Kuznetsova et al., 2020).

The material used in this study is a novel reversible photoacid-based crystalline coordination polymer. As depicted in Figure 1B, the antibacterial efficacy of this material under blue light irradiation is significantly higher than that without blue light irradiation (Liao et al., 2023). Compared with the addition of Compound 1, the antibacterial effect on *Escherichia coli* was significantly reduced when the photoacid ligand was added. This may because, although the ligand can also release protons and cause pH changes upon light irradiation, the solid nature of the photoacid ligand material affects the transfer of protons, preventing it from achieving the desired antibacterial effect. In contrast, the photoacid molecules in Compound 1 are arranged in an orderly manner, with appropriate hydrogen bonds between the proton donor and acceptor sites. This arrangement facilitates the characteristics of the photoacid and overcomes the difficulties associated with proton transfer in the excited state of solid-state photoacids (Ke et al., 2020).

In a comprehensive study involving six different bacterial strains, the material demonstrated broad-spectrum antibacterial activity, effectively inhibiting the growth of all tested strains. This finding aligns with the research conducted by Liu et al. (2021), further validating the material’s potent antibacterial properties. As illustrated in Figure 2, a comparative analysis of the addition concentrations required to achieve an 80% IR revealed a notable difference between Gram-positive and Gram-negative bacteria. Specifically, the concentration of compound 1 needed to achieve an 80% IR was higher for wild-type Gram-positive bacteria than for their Gram-negative counterparts. This discrepancy can be attributed to the thicker cell wall of Gram-positive bacteria, which confers greater tolerance to the pH changes induced by the photoacid material. This observation is consistent with the findings of Ke et al. (2020), highlighting the influence of cell wall structure on the material’s antibacterial effectiveness.

The resistance mechanism of MDR-PA involves a reduction in outer membrane permeability and an enhancement of drug efflux pumps (Wullner et al., 2022). Compared with the wild-type strains, the drug-resistant strains may have a higher tolerance to the acidic environment generated by Compound 1. MRSA possesses a unique penicillin-binding protein, PBP2a, which has a low affinity for *β*-lactam antibiotics. Compound 1 may target PBP2a, thereby affecting the structure and function of the cell membrane and cell wall, making MRSA more sensitive to pH changes. However, when the concentration of Compound 1was increased to 2 mg/mL, the IR of MRSA was lower than that of *S. aureus*. It is speculated that MRSA may have adapted to the acidic environment through self-regulation. The transcriptomic results revealed upregulation of *pbp4*, which may compensate for the cell damage caused by the downregulation of *pbp2a*. Additionally, upregulation of *infC* and *ssb* was detected, suggesting that MRSA attempts to promote protein synthesis and maintain genomic stability by increasing the expression of translation initiation factor IF-3 and single-stranded DNA-binding protein, thereby adapting to the new environment.

The effects of the material on bacterial cell surface were observed through SEM. After treatment with Compound 1, the cell surface of *E. coli*, *B. subtilis*, *P. aeruginosa*, and MDR-PA exhibited extensive shrinkage and collapse, with the permeability of the cell surface being compromised. In contrast, *S. aureus* and MRSA showed bacterial lysis and leakage of intracellular contents, indicating a disruption of cell surface integrity. These findings suggest that Compound 1 can disrupt the cell surface of the tested strains, thereby inhibiting their growth. This result is similar to the studies of Liu et al. (2021) and Zhang et al. (2022).

To further investigate the antibacterial mechanism of Compound 1, transcriptomic analysis was conducted following its addition. The results indicated that the presence of Compound 1 disrupted the synthesis and metabolic processes within bacterial cells, primarily by inhibiting the expression of related genes under acidic conditions. These genes include amino acid synthetases, cell membrane proteins, transport system proteins, and enzymes involved in oxidative stress responses.

Glutamate--ammonia ligase is an enzyme that participates in the synthesis of glutamine from glutamate and ammonia. Glutamine plays a crucial role in bacterial nitrogen metabolism, protein synthesis, and other vital processe (Gustafson et al., 1994). Aminoacyl-tRNA synthetase has a broad and profound impact on cellular protein synthesis, metabolism, and overall physiological functions. After the addition of the material, the expression levels of genes related to *glnA* and aminoacyl-tRNA synthetase (*serS*, *thrS, VXR73_RS11695*) were downregulated. This may affect the basic life activities of bacteria, such as protein synthesis and nucleic acid synthesis, thereby inhibiting the growth of MRSA.

GPX, CAT, and SOD are important ROS detoxifying enzymes in bacteria (Xu et al., 2021). When the expression of these genes is downregulated, the ability of bacteria to clear ROS is significantly reduced. This leads to lipid peroxidation of cell membranes, protein denaturation, and DNA damage. These effects disrupt the normal physiological functions of bacteria and inhibit bacterial growth (He et al., 2022; Merghni et al., 2023). In this study, after treatment with the material, the expression levels of *gpx*, *cat*, and *sod* were all downregulated, which while leading to ROS accumulation in the cells and inhibition of MRSA growth.

The Compound 1 can achieve antibacterial effects through a non-contact triggering method via blue light irradiation and a reversible proton transfer process. These characteristics offer a safer and more convenient approach for its potential clinical applications. The material demonstrates broad-spectrum antibacterial activity and shows sensitivity toward clinically relevant multidrug-resistant strains. It can be used in combination with other antibacterial agents and holds great promise for clinical antibacterial applications. However, further research is needed to explore its antibacterial mechanisms and potential safety for humans.

## 5 Conclusion

In summary, this study focused on a novel reversible photoacid-based crystalline coordination polymer and found that blue light significantly enhanced the antibacterial effect. Analysis of the IR against six pathogenic strains revealed broad-spectrum antibacterial activity, including against two clinical drug-resistant strains, with higher sensitivity toward Gram-negative bacteria. Notably, the inhibition sensitivity to MRSA was higher than that of wild-type *S. aureus*. SEM showed that the cell surface of the pathogens were damaged to varying degrees after treatment with the material, suggesting that the material disrupts cell integrity, leading to cell death. Further transcriptomic analysis revealed that after treatment with the material, bacterial protein synthesis was inhibited, the synthesis of cell membrane proteins was disrupted, and the downregulation of oxidative stress-related gene expression led to ROS accumulation, which damaged the cell membrane structure and affected normal cellular physiology and metabolism, thereby inhibiting cell growth.

## Data Availability

The datasets presented in this study can be found in online repositories. The names of the repository/repositories and accession number(s) can be found in the article/Supplementary material.

## Footnotes

^1^ http://www.bioinformatics.babraham.ac.uk/projects/fastqc/

^2^ https://ftp.ncbi.nlm.nih.gov/genomes/all/GCF/047/391/735/GCF_047391735.1_ASM4739173v1/GCF_047391735.1_ASM4739173v1_cds_from_genomic.fna.gz
