# Supplementary material for: Potential antibacterial effects and transcriptomic analysis of a novel reversible photoacid-based crystalline coordination polymer
Source: Front Microbiol. 2025 Jul 14;16:1624377. doi: 10.3389/fmicb.2025.1624377 (PMC12301377; doi:10.3389/fmicb.2025.1624377)
Supplement: Supplementary file 1 [file Data_Sheet_1.pdf]

Supplementary Material

1 Supplementary Figures

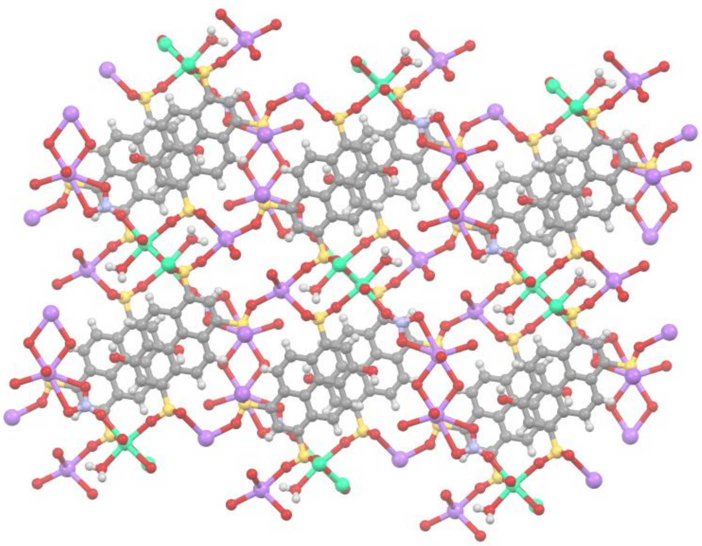

Supplementary Figure 1. The three-dimensional structure of compound **1,** the atoms represented by different color in figure: purple(Na), bright green(Ca), yellow(S), red(O), sapphire(N), dark gray(C), light gray(H).

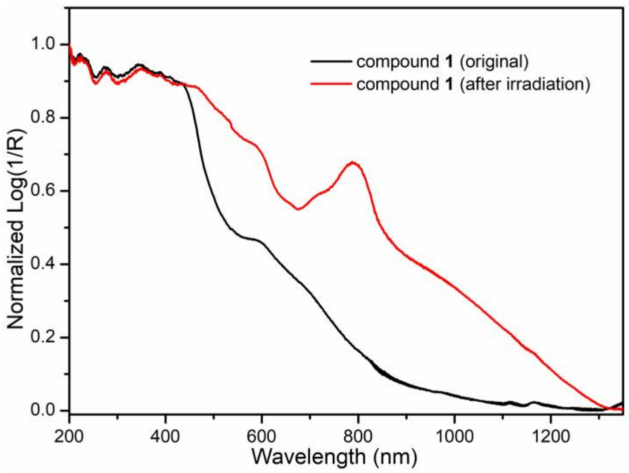

Supplementary Figure 2. UV-vis-NIR diffuse reflectance spectra for compound **1** before and after irradiation (3 W LED blue light with the wavelength of 460-465 nm).

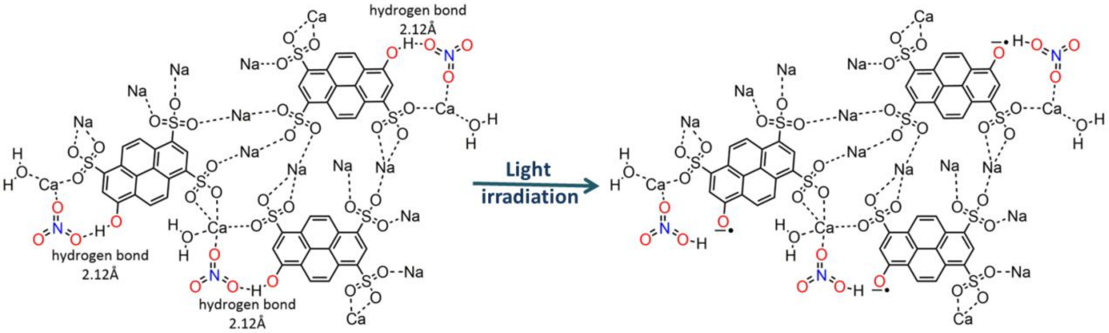

Supplementary Figure 3. Proposed mechanism for photo-induced proton transfer within crystalline compound **1**.

As previously reported by us (1), the structure of compound **1** is shown in Figure S1.The UV-vis-NIR diffuse reflectance spectra for Compound 1 before and after irradiation, shown in Figure S2, reveal that the original sample exhibits significant light absorption characteristics in the UV-visible region (200-440 nm), with additional absorption capacity in the visible-near-infrared region (440-800 nm). After light irradiation, both the light response range and absorption intensity are significantly enhanced. Specifically, the sample shows strong characteristic absorption peaks in the range of 200-465 nm, with the absorption range extended to the near-infrared region (465-1300 nm) and maintained at a high absorption intensity. Based on these light response properties, this study selected a low-power LED light source in the 400-465 nm wavelength range (corresponding to the region where the photosensitivity of Compound 1 is most significantly enhanced) to achieve efficient light regulation.

The mechanism for photo-induced proton transfer within crystalline compound **1** is depicted in Figure S3. the CCDC 2269377 contains the supplementary crystallographic data. In this system, photoexcitation not only induces an intramolecular proton transfer process but also triggers the generation of organic radicals. The synergistic action of these two photoresponsive mechanisms significantly enhances the antibacterial properties of compound **1**: proton transfer alters the charge distribution on the material's surface, promoting its interaction with bacterial cell surface (2); the continuously generated organic radicals effectively induce and disrupt the bacterial cell structure and biomolecules through their strong single-electron pairing tendency. This dual-mechanism synergistic effect enables the material to exhibit excellent broad-spectrum antibacterial activity under visible light irradiation.

2 Reference

1. Liao JZ, Liu SJ, Ke H. Excited-State Proton Transfer in a Photoacid-Based Crystalline Coordination Compound: Reversible Photochromism, Near-Infrared Photothermal Conversion, and Conductivity. Inorganic chemistry. 2023;62(41):16825-31. doi: 10.1021/acs.inorgchem.3c02271.

2. Ke H, Hu F, Meng L, Chen QH, Lai QS, Li ZC, et al. Ultrastable radical-doped coordination compounds with antimicrobial activity against antibiotic-resistant bacteria. Chemical communications (Cambridge, England). 2020;56(92):14353-6.doi: 10.1039/d0cc06379g.
